# Neutrophils in glioblastoma: orchestrators of the tumor microenvironment and immune evasion

**DOI:** 10.1007/s11033-026-11525-3

**Published:** 2026-02-03

**Authors:** Enes Demir, Elham Rahmanipour, Mohammad Ghorbani, Khushal Gupta, Maryam Zeinali, Michael Karsy

**Affiliations:** 1 School of Medicine, Eskisehir Osmangazi University, Eskisehir, Turkey; 2 Immunology Research Center, Mashhad University of Medical Sciences, Mashhad, Iran; 3 Orthopedic Research Center, Department of Orthopedic Surgery, Mashhad University of Medical Sciences, Mashhad, Iran; 4 Department of Neurosurgery, Albert Einstein College of Medicine, New York, NY USA; 5 Mazandaran University of Medical Sciences, Mazandaran, Iran; 6 Department of Neurosurgery, University of Michigan, Ann Arbor, MI USA; 7 University of Michigan Health - Ann Arbor Alfred Taubman Health Care Center, 1500 E Medical Center Dr, Ann Arbor, MI 2128, 48109 USA

**Keywords:** Glioblastoma, Neutrophils, Microenvironment, Tumor-associated neutrophils, Immunotherapy

## Abstract

Glioblastoma (GBM) is the most aggressive primary brain tumor in adults and remains refractory to current therapies. Beyond profound immunosuppression, GBM is characterized by a complex tumor microenvironment (TME) in which neutrophils have emerged as critical yet understudied regulators of tumor progression and immune evasion. Tumor-associated neutrophils (TANs) display marked functional plasticity, acquiring pro-tumor or anti-tumor phenotypes depending on microenvironmental cues. GBM recruits and reprograms infiltrating neutrophils through chemokine-driven trafficking, hypoxia, and tumor-derived cytokines, promoting angiogenesis, glioma stem-like cell support, and immune suppression via vascular endothelial growth factor (VEGF), matrix metalloproteinase-9 release (MMP-9), arginase-1, and neutrophil extracellular traps (NETs). Conversely, under inflammatory or therapeutically modulated conditions, neutrophils can exert cytotoxic and antibody-dependent anti-tumor functions and enhance T-cell responses. Clinically, elevated neutrophil-to-lymphocyte ratios and intratumoral neutrophil transcriptional signatures correlate with poor prognosis and resistance to immunotherapy. Emerging therapeutic strategies aim to modulate neutrophil recruitment, metabolism, polarization, and NET formation, often in combination with immune checkpoint blockade. This review synthesizes current knowledge of neutrophil biology in GBM, highlights their dualistic roles within the TME, and outlines translational opportunities for neutrophil-targeted therapies.

## Introduction

Glioblastoma (GBM) is the most common and aggressive primary brain tumor in adults, classified as a grade IV glioma, with an increased prevalence and incidence in the world [1–3]. The current standard of management of GBM is maximal safe surgical resection followed by radiotherapy and temozolomide-based chemotherapy [3, 4]. Nevertheless, the median overall survival remains only 14–17 months [5], with a 5-year survival rate less than 10% [6].

GBM patients demonstrate features of immunosuppression, based on decreased function of natural killer (NK) cells and T cells and high peripheral release of TGF-b (a potent immunosuppressive cytokine) and prostaglandins [7]. GBM tumor microenvironment (TME), consists of a variety of non-tumor cells and tumor cells, including immune cells, vascular cells, stromal cells, and extracellular matrix. Innate immune cells are predominant in the TME immune cells, primarily including Tumor-Associated Macrophages (TAMs), Tumor-Associated Neutrophils (TANs), NK cells, and Myeloid-Derived Suppressor Cells (MDSCs) [8].

Recent studies have shown that neutrophils can play complex roles in GBM progression, both promoting tumor growth and antitumor activity. In this regard studies evaluated neutrophil infiltration in GBM, their interaction with the TME, and their effects on tumor stage, patient survival, and treatment response. The results of these studies showed that while neutrophil infiltration may be associated with poor prognosis, certain TANs can have antineoplastic effects, suggesting dual roles. Hence, modulating neutrophil activity, through blocking infiltration, altering phenotypes, or using targeted metabolic drug delivery systems, may improve GBM therapies and patient prognosis [9].

In this narrative review, we synthesize current knowledge of neutrophil biology in the GBM TME and discuss emerging evidence linking neutrophil activity to tumor progression, therapeutic resistance, biomarkers, and therapeutic targets. Also, we provide a review of their normal functions in central nervous system (CNS) homeostasis, mechanisms of crossing the blood-brain barrier, and their role in the pathogenesis of GBM.

## Neutrophil biology and functions in the CNS

Neutrophils are short-lived innate immune cells with reported lifespan ranges from 19 h to 5.4 days [10]. They are about 70% of circulating leukocytes, and serve as rapid first-responders to infection and injury. Despite this limited lifespan, they maintain a potential for rapid deployment and functional adaptation. In favor of their extraordinary functional plasticity, they can undergo phenotypic reprogramming in response to inflammatory cytokines, metabolic cues, and tumor-derived signals, which is particularly relevant in the GBM microenvironment. In the CNS, where immune surveillance is tightly regulated, neutrophils are typically scarce under homeostatic conditions; however, during pathological states such as glioma progression, they infiltrate the CNS and exert diverse immunologic, angiogenic, and remodeling activities. This dynamic behavior underscores their importance as both early responders and modulators of downstream immune processes in brain tumors. They have remarkable plasticity, adapting their phenotype to local signals. TANs have been described to polarize into N1-like (anti-tumor) and N2-like (pro-tumor) states based on TME exposure and interaction with glioma cells [8]. However, this N1/N2 framework should be regarded as a useful but exploratory conceptual model rather than a strict binary classification, as emerging single-cell and spatial transcriptomic data suggest that GBM-infiltrating neutrophils occupy a continuum of transcriptional and functional states shaped by cytokines, hypoxia, metabolic cues, and spatial niche.

N1-like TANs exhibit a hypersegmented nucleus, and its phenotype is characterized by high expression levels of Fas, tumor necrosis factor-α (TNF-α), and intercellular adhesion molecule-1 (ICAM-1) [8, 11]. Notably, depletion of N1-like TANs has been shown to promote tumor growth. N1-like TANs play anti-tumor roles early in tumorigenesis via phagocytosis, reactive oxygen species (ROS)-mediated cytotoxicity, Fas-ligand-mediated apoptosis, antibody-dependent cellular cytotoxicity, enhanced antigen-presenting capacity, and elevated production of cytokines that promote antitumor immune responses [8, 11, 12]. However, prolonged exposure to glioma-derived factors such as IL-6, IL-8, IL-1β shifts them toward a pro-tumor N2-like state, increasing angiogenesis, invasion, drug resistance, and immunosuppression [8, 12].

**Fig. 1:**
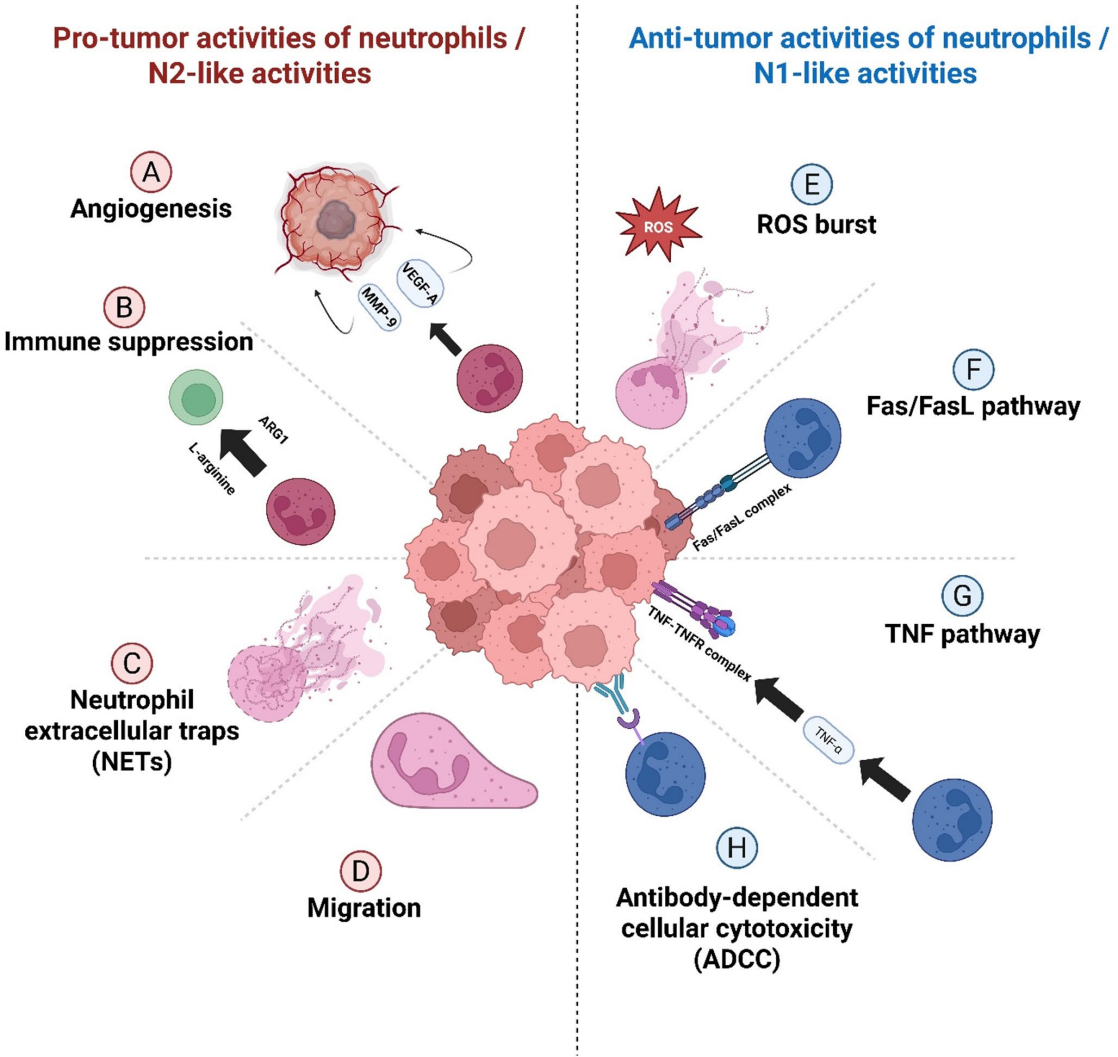
Dual roles of tumor-associated neutrophils (TANs) in glioblastoma. Schematic overview of the pro-tumor (N2-like) and anti-tumor (N1-like) activities of neutrophils within the glioblastoma tumor microenvironment. **(A)** *Angiogenesis*: N2-like TANs promote aberrant neovascularization through the release of vascular endothelial growth factor (VEGF-A) and matrix metalloproteinase-9 (MMP-9), facilitating endothelial sprouting and extracellular matrix remodeling. **(B)** *Immune suppression*: TANs suppress adaptive immunity through arginase-1 (ARG1)–mediated depletion of L-arginine, resulting in impaired T-cell proliferation and effector function. **(C)** *NETs*: Formation of NETs composed of chromatin, proteases, and reactive oxygen species (ROS) promotes tumor invasion, vascular permeability, blood–brain barrier disruption, and immune evasion. **(D)** *Migration*: Chemokine-driven neutrophil trafficking and retention within hypoxic and perivascular niches support tumor infiltration, spatial organization, and sustained pro-tumor signaling. Conversely, under inflammatory or therapeutically modulated conditions, neutrophils can exert anti-tumor functions. **(E)** Reactive Oxygen Species (*ROS) burst*: N1-like TANs generate high levels of ROS that directly induce tumor cell damage and apoptosis. **(F)** *Fas/FasL pathway*: Neutrophil-expressed Fas ligand (FasL) engages Fas on glioblastoma cells, triggering extrinsic apoptotic signaling. **(G)** Tumor necrosis factor (*TNF) pathway*: TNF-α released by activated neutrophils induces TNF receptor–mediated cytotoxic signaling in tumor cells. **(H)** *ADCC*: Fcγ receptor–mediated engagement of antibody-opsonized tumor cells enables neutrophil-driven cytotoxicity and tumor cell elimination. Together, this figure highlights the functional plasticity of TANs in glioblastoma and the balance between tumor-promoting and tumor-restraining programs shaped by the local microenvironment. The figure was created using BioRender (software version 04)

N2-like TANs are characterized by a circular nucleus and express high levels of *CXCR4*, *arginase-1* (*ARG1*), *VEGF*, and *MMP-9*, which promote tumor growth and suppress the antitumor immune responses [8, 12]. Targeting TANs may represent a novel therapeutic strategy, potentially enhancing the efficacy of existing cancer immunotherapies and other treatment modalities [8, 12]. Despite the existence of functional differences, no definitive surface markers have been identified to distinguish N1-like and N2-like TANs. The dual pro-tumorigenic and anti-tumorigenic roles of tumor-associated neutrophils in glioblastoma are summarized schematically in Fig. 1. Consistent with this, high-resolution single-cell and spatial profiling studies increasingly indicate that neutrophils in tumors, including GBM, do not segregate into discrete N1 and N2 populations but instead span a dynamic spectrum of intermediate and hybrid activation states. These states appear to be highly context-dependent and spatially restricted, reflecting local gradients of hypoxia, interferons, TGF-β, and metabolic stress within the tumor microenvironment. Further studies to distinguish the N1/N2 classification of neutrophils are needed to understand tumor biology and target tumors.

In normal physiological conditions, immune cells are rarely found in the brain parenchyma, where the blood-brain barrier (BBB) and blood-cerebrospinal fluid barrier (BCSFB) can prevent immune cells and molecules from entering the brain. Neutrophils infiltrate the CNS during inflammation and tumors [8, 13]. Thus, neutrophils do not routinely traverse an intact BBB but instead access the CNS through regions of barrier dysfunction and specialized routes such as the choroid plexus under pathological conditions [8, 13]. CNS-infiltrating neutrophils may arise from bone marrow in the skull and adjacent vertebrae, rather than distant marrow [8, 14]. Herrison et al. demonstrated microscopic vascular channels connecting the cranial bone marrow cavity to the dura mater in both murine and human craniotomy specimens [15, 16]. Neutrophils migrate to the brain through close storage areas. Neutrophils attracted by chemotactic factors in the TME, infiltrate the TME through ChP under the attraction of CXCL1/2. Cytokines released by neutrophil extracellular traps (NETs) can disrupt the integrity of the BBB and facilitate the infiltration of neutrophils into the brain parenchyma [8].

## Neutrophils in the GBM tumor microenvironment (TME)

GBM establishes strong chemokine gradients that recruit circulating neutrophils into the tumor through regions of blood–brain barrier (BBB) or blood–tumor barrier (BTB) disruption and perivascular niches rather than by traversal of an intact BBB [8]. Key axes include CXCL8/IL-8, CXCL1/2→CXCR2, and CXCL12→CXCR4; these signals are amplified by tumor-educated myeloid cells and endothelial niches, driving perivascular accumulation and parenchymal entry of TANs [8]. Within the GBM milieu, tumor and myeloid cells secrete cytokines (e.g., TNF-α, IL-6) and alarmins (S100A8/A9) that both recruit and “imprint” infiltrating neutrophils. This milieu upregulates neutrophil activation markers (CD11b, CD66b), modulates chemokine receptors (CXCR1/2/4), and sustains survival, thereby enriching an immunosuppressive, pro-angiogenic TAN phenotype localized to the perivascular niche. The central role of the CXCR2 axis in neutrophil trafficking was evaluated in vitro showing tumor-derived IL-8 and related ligands trigger neutrophil chemotaxis toward tumor spheroids, while pharmacologic CXCR2 blockade (e.g., AZD-5069) reduces migration. Although demonstrated in another solid tumor context, these mechanisms are conserved and highlight why CXCR2 signaling is a prime target for limiting neutrophil ingress into malignant brain tissue [17]. Additional tumor-derived cues relevant to GBM, granulocyte colony-stimulating factor/colony stimulating factor 3 (G-CSF/CSF3), hypoxia/hypoxia-inducible factor-1 (HIF-1), driven chemokines, and NETosis-linked mediators (e.g., IL-8, HMGB1) further potentiate recruitment and infiltration. These signals not only increase the flux of neutrophils into GBM but also facilitate their retention and functional reprogramming, reinforcing immunosuppression and vascular remodeling within the TME [18].

Neutrophil phenotype is geographically constrained. TANs are also observed to cluster in perinecrotic and perivascular areas with hypoxia, alarmins, and lactate [8, 19]. Hypoxia polarizes them for the expression of *VEGF-A* and *MMP-9* for angiogenesis promotion and remodeling. Spatial transcriptomics reveal GBM-infiltrating neutrophils express *VEGFA*,* THBD*,* ICAM1*,* S100A9*, and *MMP-9*, which are associated with edema and aggressive growth. But not all niches are so suppressive: perivascular crevices of higher oxygen or tolerable antibody-access zones may briefly harbor N1-like activities, e.g., antibody-dependent cellular cytotoxicity (ADCC). Single-cell/spatial assays reveal heterogeneity between zones and temporally; neutrophils can change when tumors change, or therapy makes interferon-rich milieus. Location-sensitive assays (e.g., perivascular vs. invasive edge counts) are consequently more informative than bulk [8, 19].

GBM TME is spatially heterogeneous with compartmentalization of hypoxia, chemokine gradients, and cell–cell interactions which impact myeloid subtypes along the tumor from core to margin [20, 21]. Topologies of myeloid patterns correlate with outcome, with outcome, microglia-enriched, margin-oriented niches tend to associate with better survival, whereas core-dominant, bone-marrow–derived macrophage/MDSC programs track with worse prognosis. Multi-regional single-cell profiling shows fragment-to-fragment variation in glioma and immune populations, microglia versus bone-marrow derived macrophage balance, and ligand–receptor programs, demonstrating intra-/inter-tumoral and temporal differences across newly diagnosed and recurrent GBM [22].

Neutrophils interface with monocyte-derived macrophages (MDMs) and microglia across GBM niches, necrotic cores, perivascular/perinecrotic regions, and invasive edges, creating spatially organized dialogue that shapes recruitment, phenotype, and effector programs [23]. Neoplastic–myeloid reciprocity induces mesenchymal states in tumor cells and remodels myeloid expression profiles, reinforcing pro-tumor functions and heterogeneity within the TME [23]. Treg depletion further rewires this network by boosting IFN-γ–dependent activation and FcγR upregulation in intratumoral myeloid cells, expanding FcγR^hi^ MDMs and enhancing phagocytosis, while coordinating with CD8 + T-cell responses [24]. This multi-cell crosstalk positions neutrophils and macrophage/microglia subsets as co-orchestrators of immune evasion and therapeutic responsiveness in GBM [23, 24].

Neutrophils in the GBM microenvironment are significantly influenced by their interaction with T cells and other myeloid cells. Specifically, depleting Tregs in mouse GBM models increases intratumoral IFN-γ levels, which, in turn, boosts myeloid cell activation [24]. This activation includes the upregulation of FcγR expression on neutrophils and macrophages, making them more effective at phagocytosis and ADCC against cancer cells opsonized with antibodies. Furthermore, combining Treg depletion with anti-PD-1 therapy and an anti-tumor antibody (such as anti-EGFRvIII) leads to improved tumor control. This suggests that Fc-engaged neutrophils and macrophages are critical executioners in this therapeutic strategy. Beyond direct anti-tumor activity, IFN-γ-activated neutrophils also contribute to the adaptive immune response by recruiting T cells via chemokines and by supplying antigens to dendritic cells for processing. Therefore, clinical approaches that either promote a Th1 immune response or inhibit Treg-mediated suppression hold promise for steering neutrophils toward anti-tumor functions in GBM.

## Pro-tumorigenic functions of neutrophils in GBM

GBM-associated neutrophils cluster perivascularly near proliferating endothelial cells and associate with deformed vessels, while their transcriptomes are enriched for angiogenesis programs with upregulation of *VEGFA/THBD/ICAM1* [25]. Additionally, TANs are a major intratumoral source of MMP9, a VEGF-linked protease released extracellularly to degrade basement membrane components, enabling both angiogenic sprouting and tumor cell invasion. These coordinated activities amplify aberrant neovascularization and create a microenvironment conducive to rapid tumor growth, implicating TAN-driven angiogenesis as a critical contributor to GBM progression [25–27].

Tumor-infiltrating neutrophils (TINs) representing the intratumoral subset of TANs in glioma, correlate positively with *PD-L1* expression and poorer outcomes [28]. Dual targeting of PD-1 and neutrophil depletion has shown improved tumor control and survival in intracranial animal models. TINs directly dampen adaptive and innate anti-tumor immunity by suppressing T cells and NK cells; neutrophil-enriched transcriptional programs are prominent in GBM nonresponders to anti-PD-1, implicating TINs in checkpoint resistance [28]. Mechanistically, TAN-mediated resistance to immune checkpoint blockade likely involves protease-mediated destruction of T cells (attractant chemokines, arginase-dependent T-cell suppression, NET), induced physical and signaling barriers, and reinforcement of immunosuppressive cytokine loops [29].

Beyond their intratumoral localization, neutrophil populations with polymorphonuclear myeloid-derived suppressor cell (PMN-MDSC)–like features further reinforce immune evasion by highly expressing arginase-1 and suppressing T-cell proliferation and IFN-γ production, thereby creating a metabolically driven brake on T-cell function [23, 30, 31]. In hypoxic GBM niches, neutrophils undergo metabolic reprogramming toward a CD71⁺ subset characterized by lactate-induced histone lactylation that upregulates arginase-1 and potentiates robust T-cell suppression; inhibition of this pathway reverses immunosuppression and enhances tumor sensitivity to immunotherapy [30]. Conceptually, monocyte–neutrophil crosstalk and broader myeloid plasticity in the GBM microenvironment reinforce these immunosuppressive circuits and shape responses to standard and emerging immunotherapies [23].

Within the TAN, cytokine dynamics dictate the balance between N1 and N2 states. Specifically, type I/II interferons (IFN-β/IFN-γ) and granulocyte–macrophage colony-stimulating factor (GM-CSF) promote N1 characteristics, whereas TGF-β favors N2 polarization [8, 11]. Interestingly, IFN-β can repolarize N2-like TANs to an N1 phenotype. Consequently, N1 programs only develop in GBM when powerful inflammatory signals, like those present during successful immunotherapy, intercurrent infections, or periods of temporary reduction in IL-6/IL-8 levels, are sufficient to overcome the tumor’s N2-polarizing influence [8].

Neutrophils foster GBM stem-like niches by expanding the glioma stem cell pool through S100A4-dependent signaling, linking TAN influx with stemness and tumor progression [26]. In vitro, neutrophil co-culture or conditioned media increases glioma-initiating cell proliferation and survival and motility and induces a mesenchymal shift with increased *YKL-40/MMP2* and decreased *Nestin* expresssion [32]. Functionally, S100A4 knockdown blunts neutrophil-driven invasion/mesenchymal programming and enhances anti-VEGF efficacy, underscoring TAN support of stem-like niches [32].

TANs also promote tumor progression through the formation of NETs, defining webs of chromatin, DNA and protein involved in pathogen targeting [8]. NETs are decorated with myeloperoxidase (MPO), ROS, neutrophil elastase (NE), and MMP-9, which can disrupt BBB tight junctions (claudin-5/occludin/ZO-1), increase vascular permeability, and facilitate further TAN influx into the necrotic GBM core, accelerating invasion and angiogenesis [8]. NET modification by high mobility group protein 1 (HMGB1) activates RAGE–NF-κB signaling in GBM cells, boosting IL-8-CXCR2-mediated ROS generation and NETosis [33]. This can form a self-reinforcing positive feedback loop. NET enzymes (e.g., NE, MMP-9) can cleave cytoskeletal matrix (e.g., laminin) to impede dendritic/T-cell function while promoting neutrophil infiltration, foster immune evasion, and worsen tumor progression. Augmenting pharmacologic NET blockade (e.g., DNase I, protein arginine deiminase 4 (PAD4) inhibition, anti-HMGB1) may be a rational therapeutic strategy in GBM and is currently under investigation [8, 34].

## Anti-tumor roles of neutrophils

Although tumor-associated neutrophils in glioblastoma predominantly acquire immunosuppressive and pro-tumorigenic programs, accumulating evidence indicates that neutrophils retain latent and inducible anti-tumor capacities that can be unmasked under inflammatory or therapeutically modulated conditions. These anti-tumor functions are most evident during early tumor development, acute infections, vaccine-like immune stimulation, or effective immunotherapy, when interferon-rich and Fc-engaging microenvironments transiently override dominant N2-like polarization cues. Importantly, these observations suggest that neutrophils are not irreversibly committed to tumor-promoting roles but instead represent a plastic effector population that can be redirected toward tumor-restraining activity.

While neutrophils are usually involved in supporting the tumor, they also possess their own intrinsic effector functions that can kill glioma cells with their classical cytotoxic programs. They produce ROS through NADPH oxidase, which can induce cytotoxic Ca²⁺ entry by tumor-cell transient receptor potential melastatin 2 (TRPM2) channels and activate apoptosis by Fas ligand (FasL)–death receptor interaction [8, 34]. Neutrophils also phagocytose tumor remains and perform ADCC upon opsonization by antibodies targeting glioma cells. Fcγ receptors, particularly FcγRIIa, bind IgG, promoted degranulation, and induce a membrane-lesioning mode of killing termed “trogoptosis”. Granule proteases and TNF-related apoptosis-inducing ligand (TRAIL) expressed on neutrophils engage TRAIL death receptors (DR4/DR5) on glioma cells, thereby directly activating extrinsic (caspase-8–dependent) apoptosis and inducing secondary killing in glioma cells. These mechanisms work best in acutely inflamed environments (e.g., infections, vaccine-like stimulation, early tumor phase), uncovering a latent anti-tumor defense potential which GBM sets the stage to suppress [8, 34].

Collectively, these findings underscore that neutrophils possess a substantial but context-dependent tumor-restraining potential in GBM. While these effector programs are typically silenced by hypoxia, TGF-β signaling, metabolic reprogramming, and immunosuppressive cytokine gradients within the tumor microenvironment, they can be re-engaged by interferon signaling, Fcγ receptor ligation, and relief of metabolic constraints. This duality provides a strong mechanistic rationale for therapeutic strategies aimed at reprogramming, rather than depleting, tumor-associated neutrophils to restore cytotoxicity, antibody-dependent cellular cytotoxicity, and cooperative interactions with T cells and macrophages.

## Clinical correlations and prognostic implications

### Systemic inflammation markers

The neutrophil-to-lymphocyte ratio (NLR) is a reliable, simple systemic biomarker with prognostic significance in GBM. An elevated pretreatment NLR accurately predicts poorer overall survival; meta-analysis (~ 1,200 patients) estimates ~ 1.6 hazard ratio for death with rising NLR [8]. Elevated pretreatment NLR values consistently correlate with reduced overall survival, increased tumor aggressiveness, and enhanced myeloid-skewed immune activity in multiple retrospective and prospective studies [10, 35, 36]. Changes in NLR during therapy may also provide insights into treatment response or recurrence patterns, suggesting potential utility as a dynamic biomarker [37]. Though tempting for risk stratification and trial enrichment, clinical utility depends on standardized cut-offs and adjustment for confounders such as infections and steroids [8, 38].

Beyond the ratio itself, overt neutrophilia (elevated absolute neutrophil counts) is a recognized systemic feature of glioma and GBM. Expression of G-CSF and GM-CSF by astrocytoma and glioma cells provides a tumor-derived myelopoietic drive [39]. Patients with brain tumors also display elevated circulating IL-6, IL-1β, TNF-α, IL-8, VEGF, and GM-CSF, cytokines associated with tumor progression and systemic inflammation [40]. These mediators skew hematopoiesis toward granulopoiesis and contribute to tumor-driven granulocytosis and neutrophilia with relative lymphopenia, mechanistically underpinning the high NLR observed in many GBM patients. Clinically, pronounced neutrophilia during the disease course often tracks with more aggressive disease and shorter survival. Thus, NLR acts as a surrogate of GBM-driven myeloid bias rather than a nonspecific inflammatory signal [8, 38].

### Transcriptomic and spatial signatures

Bulk analyses (e.g., The Cancer Genome Atlas) detect neutrophil-enriched modules, CXCL8/CXCR2 axis, S100A8/A9, NETosis genes (MPO, NE, PAD4, MMP9), that are common in mesenchymal GBM, a hypoxic, inflamed subgroup with dense myeloid infiltration. Single-cell RNA-seq finds heterogeneous neutrophil/PMN-MDSC states in the face of technical hurdles such as clusters outlined by S100A8/A9, ARG1, IL-8, and CXCR2. Spatial profiling plots out neutrophil-dense areas to perinecrotic, immunosuppressive niches with high PD-L1 and TGF-β and associates these patterns with poorer survival [8]. Hence, prognosis cannot merely reflect neutrophil presence but their program and location, angiogenesis/immunosuppression versus cytotoxicity. The combination of NLR with intratumoral transcriptomic/spatial features may possibly enhance risk prediction.

### Polymorphonuclear MDSCs (PMN-MDSCs) in blood and tumor

There is a considerable percentage of GBM-associated neutrophils that are PMN-MDSCs, low-density, ARG1- and S100A8/A9-high cells that have strong T-cell suppressive activity in blood and tumors [29]. ARG1 depletes L-arginine from the extracellular milieu, down-regulating CD3ζ and blunting T-cell proliferation; inhibition or supplementation of arginase or arginine reverses in vitro suppression [41]. Hypoxia augments this phenotype. Recent research defines a hypoxia-inducible, CD71⁺ subpopulation of neutrophils in GBM with augmented glycolysis, lactate production, and histone lactylation that up-regulates ARG1 to generate highly suppressive cells; genetic or pharmacologic deletion of ARG1 eliminates their T-cell suppression. Neutrophil lactate-induced lactylation inhibition reduced ARG1, eliminated T-cell suppression, and re-established PD-1 checkpoint sensitivity in GBM models [42]. These findings suggest metabolic re-patterning, most importantly the hypoxia–lactate–ARG1 axis, as a highly manipulable vulnerability. NLR optimization with clinical biomarkers like plasma S100A8/A9, ARG1 activity, or histone lactylation signatures could identify patients maximally loaded with suppressive neutrophils.

A multi-layered approach is needed for GBM risk stratification. Systemically, the NLR indicates myeloid-skewed inflammation. Within the tumor, neutrophil density and distribution reveal their role in driving angiogenesis and immune escape. Furthermore, transcriptomic and metabolic markers such as high CXCR2 ligands, S100A8/A9, ARG1, and NETosis signatures identify dominant suppressive mechanisms. Integrating these layers can improve prognosis and guide the selection of patients who would benefit from adding neutrophil-targeted agents to immune checkpoint blockade.

## Therapeutic implications and emerging strategies

### Curbing neutrophil recruitment and tissue entry

GBM released CXC chemokines (CXCL8/IL-8, CXCL1/2) can recruit CXCR2⁺ neutrophils and utilize CXCL12 for attracting CXCR4⁺ cells [19]. AZD-5069, a CXCR2 antagonist, inhibited IL-8–mediated GBM chemotaxis and tumor spheroid invasion in preclinical models [42]. Targeting the CXCL8/CXCR2 axis, key to TAN recruitment in GBM, via CXCR2 inhibition holds promise for reducing intratumoral neutrophil influx and attenuating neutrophil-mediated tumorigenesis, angiogenesis and immunosuppression. Although these data derive primarily from non-CNS tumor systems, the conservation of CXCR2 signaling mechanisms across solid tumors suggests a strong rationale for evaluating such inhibitors in GBM. Early-phase trials or window-of-opportunity studies in GBM could help establish feasibility, pharmacodynamics, and intratumoral target engagement.

Inhibition of CXCR4 via a drug such as plerixafor is an alternative, but one-target activity elsewhere in the body, such as hematopoiesis, needs caution. Early studies can employ window-of-opportunity designs (pre-resection treatment) to demonstrate intratumoral on-target effects: decreased TAN density and inhibition of CXCR2-responsive gene signatures, validated by imaging or PET tracers to confirm brain penetration. Infection monitoring is necessary with neutrophil trafficking disruption [42]. Local delivery via convection-enhanced catheters or biodegradable wafers is employed to deliver high intratumoral exposure with low systemic immunosuppression. Due to chemokine redundancy, combination regimens such as dual CXCR2/CXCR4 inhibition will be required.

### Reprogramming the neutrophil phenotype and metabolism

Reprogramming, rather than depletion, of intratumoral TANs into an anti-tumor or neutral state has been evaluated as a treatment option, reflecting a shift toward strategies that preserve essential innate immune functions while redirecting neutrophil activity within the GBM microenvironment. This approach leverages the inherent plasticity of TANs, which can adopt pro- or anti-inflammatory phenotypes depending on cytokine exposure, metabolic cues, and hypoxic stress, and provides a framework for therapies aimed at converting suppressive N2-like cells into more inflammatory, cytotoxic N1-like counterparts [8, 11, 27].

#### Hypoxia–lactate–ARG1 axis

Hypoxia activates neutrophils to glycolysis and lactate accumulation; lactate-dependent histone lactylation is an ARG1 expression regulator and a T-cell inhibitor [43]. Lactate-dependent histone lactylation functions as an epigenetic regulator that drives elevated ARG1 expression, thereby enhancing the immunosuppressive phenotype of TANs and directly inhibiting T-cell proliferation and effector function [30, 43]. This hypoxia–lactate–ARG1 axis represents a critical metabolic vulnerability, as pharmacologic blockade of lactate production, histone lactylation, or ARG1 activity has been shown to reverse neutrophil-mediated T-cell suppression and restore responsiveness to anti–PD-1 therapy in preclinical GBM models [30]. These findings highlight the potential for metabolic reprogramming strategies to synergize with immunotherapy in otherwise treatment-resistant tumors. Pharmacologic compounds that diminished lactate production or lactylation (p300/CBP-mediated acylation inhibition) diminished ARG1 in preclinical GBM, re-established local T-cell suppression reversal, and sensitized the tumor to PD-1 blockade [30]. Endpoints include decreased neutrophil ARG1 activity, decreased lactylation at the ARG1 locus, and augmented intratumoral IFN-γ/IL-2.

#### Lifespan/polarization signals

Neutrophil polarization in GBM is tightly regulated by cytokines that shape their survival, activation state, and effector programs within the TME. Tumor-derived IL-6 and IL-8 sustain neutrophil viability by delaying apoptosis and reinforcing N2-like, pro-tumorigenic features through STAT3 and NF-κB signaling, promoting expression of ARG1, VEGF, and MMP-9 and supporting angiogenesis, immune suppression, and matrix remodeling [8, 18, 42]. Consequently, pharmacologic disruption of these pathways—via IL-6R blockade with tocilizumab-like agents or CXCR1/2 antagonism such as AZD-5069 or reparixin—has the potential to shorten the lifespan of N2 neutrophils, interrupt their recruitment, and bias infiltrating TANs toward more inflammatory, N1-like phenotypes [17, 29, 42].

TGF-β represents another dominant suppressive cue in GBM, acting as a master regulator that shifts neutrophils toward N2 states by repressing pro-inflammatory cytokines, diminishing cytotoxicity, and increasing pro-angiogenic output [11, 27]. Blockade of TGF-β signaling with drugs such as galunisertib or fresolimumab can restore N1-associated features, such as TNF-α and ICAM-1 expression, while reducing ARG1, VEGF, and CXCR4-driven retention, as shown in preclinical cancer models and supported indirectly by GBM immune-modulatory studies [8, 11, 27]. Importantly, the outcome of manipulating these cytokine circuits may depend on hypoxic gradients and metabolic reprogramming—particularly lactate-driven histone lactylation, a potent inducer of ARG1 and immunosuppressive programming—highlighting the need for integrated strategies that target both cytokine and metabolic axes [30, 43].

#### Ecosystem reset

Limited Treg modulation as a result of low-dose anti-CD25 or agents blocking Treg function can increase intratumoral IFN-γ/GM-CSF, elevating neutrophil FcγR and facilitating ADCC when in combination with tumor-specific antibodies [24]. The goal is to retain healthy neutrophil functions such as phagocytosis, debris clearance, and ADCC while deleting suppressive/angiogenic outputs.

### Disrupting NETs and their feedback loops

Given the pro-tumor functions of NETs in BBB disruption, vascular leak, matrix remodeling, and immune exclusion, pharmacologic disruption of NET structures and signaling loops is an attractive strategy in GBM. Recombinant DNase I (e.g., dornase alfa), which breaks down extracellular DNA, can therapeutically dissolve NETs in secondary diseases and suppresses pro-metastatic NET functions in cancer models [44]. PAD4 inhibitors (e.g., chloramidine) inhibit histone citrullination and NET formation, functionally mirroring DNase I in reducing NET burden and re-establishing immune surveillance [44]. Inhibition of HMGB1–RAGE signaling disrupts the NET-IL-8 loop and dampens NET-mediated inflammation [33].

### Drug delivery and safety in the CNS

BBB and BTB limit the entry of CNS drugs, especially large or hydrophilic molecules. Direct methods, convection-enhanced delivery to resection and tumor cavities, intracavitary wafers, can deliver therapeutics to TANs with minimal systemic impact. Nanoparticles or conjugates (e.g., LDL- or transferrin-coated) could facilitate brain entry and targeting to cells. Bispecific antibodies that target the brain matrix with activation of neutrophil receptors are an appealing concept. Although the BTB is partially disrupted in GBM, permeability varies spatially across the tumor and remains largely intact in infiltrative margins where therapeutics are most needed. These anatomical constraints complicate the delivery of neutrophil-modulating therapies—such as chemokine receptor antagonists, peptidylarginine deiminase isozyme-specific inhibitors, or metabolic modulators—and frequently necessitate alternative approaches such as convection-enhanced delivery, biodegradable wafers, or nanoparticle-based formulations [45].

GBM patients also commonly develop drug-induced lymphopenia; use of neutrophil-targeting drugs is hazardous for infection and wound-healing complications. Intermittent or local dosing and perioperative timing are prudent approaches. Trials must show intratumoral pharmacodynamics (e.g., decreased TANs or NET markers; decreased CXCR2-responsive transcripts) and, if possible, intrathecal or intraoperative bioavailable drug levels. Imaging and investigational PET tracers such as MPO, IL-8 can assess target engagement noninvasively. Potential pharmacodynamic biomarkers include circulating NET complexes (DNA–MPO, citrullinated H3) and radiographic edema. Effective NET disruption would be expected to correlate with increased intratumoral lymphocyte infiltration and reduced immunosuppressive myeloid features [44]. As NETs enable host defense and thrombosis, intermittent or localized approaches (e.g., peri-operative PAD4 inhibition or intracavitary DNase) may help balance anti-tumor efficacy with preservation of systemic innate immunity.

Taken together, the therapeutic strategies discussed conceptually map distinct neutrophil functions in GBM—including recruitment and tissue entry, immunosuppression, neutrophil extracellular trap (NET) formation, metabolic reprogramming, and inducible anti-tumor effector activity—to corresponding intervention approaches. These include blockade of neutrophil trafficking pathways, inhibition of NET formation, targeting of immunosuppressive and metabolic programs, and phenotype reprogramming strategies aimed at restoring cytotoxicity and cooperative interactions with adaptive immunity. This integrative framework highlights how mechanistic insights into neutrophil biology in GBM can be directly translated into rational therapeutic design.

### Underexplored clinical modifiers

Several clinically relevant modifiers of neutrophil biology in glioblastoma remain underexplored. Standard-of-care therapies such as radiotherapy and temozolomide profoundly remodel the tumor microenvironment and are therefore likely to influence neutrophil recruitment, polarization, and effector function in ways that remain poorly characterized. Host-related variables, including sex and age, as well as systemic factors routinely encountered in GBM management—particularly corticosteroid exposure—may further shape tumor-associated neutrophil phenotypes and confound immunological readouts. Systematic integration of these dimensions into future experimental and clinical studies will be essential for accurately defining neutrophil function and for translating neutrophil-modulating therapies into clinical practice.

## Knowledge gaps and future directions

Despite accumulating evidence that neutrophils are crucial players in shaping the GBM microenvironment—driving angiogenesis, supporting glioma stem-like programs, reinforcing immune evasion, and contributing to therapy resistance through chemokine-guided recruitment, hypoxia-driven reprogramming, metabolic rewiring, and NET-mediated matrix remodeling—substantial gaps remain in our understanding of how these highly plastic cells diversify into context-dependent phenotypes across the heterogeneous and evolving landscape of GBM. In particular, the field still lacks a unified framework to define neutrophil heterogeneity, resolve spatially restricted functional states, and characterize the temporal dynamics by which circulating, bone-marrow–derived, and CNS-resident neutrophil subsets emerge, interact with macrophage/microglia networks, influence T-cell function, and adapt under therapeutic pressure. Addressing these gaps through high-resolution phenotyping, longitudinal and spatially informed profiling, and translational strategies that consider CNS-specific constraints will be essential for converting neutrophil biology from a mechanistic insight into an actionable therapeutic axis in GBM.

### Heterogeneity of neutrophil subpopulations and the need for deeper phenotyping of neutrophil subpopulations in GBM

Studies treated poorly defined TANs as a homogenous population of short-lived effector cells, yet emerging data suggest that neutrophils are highly plastic immune cells capable of acquiring context-dependent phenotypes ranging from pro-tumorigenic to potentially anti-tumorigenic states [8, 11, 14, 27, 46–48]. Recent work describes transcriptionally and functionally distinct neutrophil subsets—including pro-angiogenic and immunosuppressive populations, hybrid antigen-presenting neutrophils, and more cytotoxic or inflammatory states—highlighting the substantial heterogeneity of TANs in GBM [11, 14, 25, 30, 46, 47]. Despite these advances, no consensus framework exists for defining neutrophil states in human tumors, as phenotypic boundaries remain fluid and context-dependent [27, 47, 48]. This emerging diversity likely reflects the influence of hypoxia, cytokine gradients, glioma stem-like cell interactions, and local metabolic cues, all of which dynamically reprogram neutrophils across GBM niches [8, 21, 30, 32, 43]. A deeper understanding of TAN subsets is essential to identify which populations drive tumor progression and which may be redirected toward anti-tumor functions.

Canonical surface markers (e.g., CD15, CD16, CD66b) inadequately capture the diversity of neutrophil subsets, and functional dichotomies such as “N1 (anti-tumor)” versus “N2 (pro-tumor)” remain overly simplistic. It is unclear whether GBM harbors transcriptionally and functionally distinct subsets analogous to low-density neutrophils, immature myeloid-derived suppressor cells, or specialized pro-angiogenic populations described in other cancers. A systematic characterization of these subpopulations in GBM is lacking. Bulk profiling masks functional heterogeneity, and the field lacks a consensus framework to distinguish pro- versus anti-tumor subsets within GBM. High-dimensional approaches, including mass cytometry and advanced flow cytometry, could help delineate neutrophil subsets based on surface markers, functional signatures, and metabolic states. Such nuanced phenotyping may reveal clinically relevant subgroups that represent stable lineages, transient activation states, or context-dependent reprogramming induced by the tumor milieu and correlate with prognosis or treatment response.

### Single-cell and Spatial transcriptomic approaches

While bulk transcriptomic analyses have provided insights into neutrophil-associated gene expression, they fail to capture intratumoral heterogeneity and spatial context. Single-cell RNA sequencing has already revolutionized the study of T cells and macrophages in brain tumors, but its application to neutrophils has been limited by their fragile nature and low RNA content. Emerging optimized single-cell RNA sequencing, coupled with spatial transcriptomics, offers the opportunity to resolve this complexity by mapping neutrophil states at high resolution and to understand how they interact with other immune and stromal cells within the tumor microenvironment [48–55]. Integrating these approaches with imaging-based methods could uncover microanatomical perivascular niches, hypoxic gradients, migratory routes, crosstalk with glioma stem-like cells, metabolic adaptations, and the molecular cues driving their polarization in GBM.

### Dynamic and longitudinal analyses in patients and preclinical models

Current studies rely on static tumor samples, offering only snapshots of neutrophil biology at diagnosis or recurrence. However, GBM is a highly dynamic disease shaped by tumor evolution, therapeutic pressure, and systemic immune responses. Longitudinal studies that track the dynamic reprogramming of neutrophil populations over time during progression, recurrence, and therapy —both in patients and in preclinical models—are essential to capture these dynamics [56–58]. Serial sampling of blood, cerebrospinal fluid, and, when feasible, tumor tissue could provide valuable insights into how neutrophil recruitment, activation, and functional states shift across disease progression and treatment [59–61]. In preclinical models, lineage-tracing strategies and intravital imaging may reveal the temporal kinetics of neutrophil infiltration, their interactions with tumor vasculature, and their contribution to therapy resistance [62, 63]. Such studies may also uncover biomarkers that predict response to treatment or disease progression.

### Translational barriers in targeting neutrophils in brain tumors

Although neutrophils are attractive therapeutic targets, translation into clinical utility faces unique challenges [64, 65]. First, their essential roles in host defense, antimicrobial defense, wound healing, and tissue homeostasis raise concerns about systemic immunosuppression when depleting or broadly inhibiting them. The blockade of chemokine axes such as CXCL8–CXCR1/2 may disrupt essential inflammatory responses. Systemic inhibition of their recruitment or effector functions risks compromising host immunity and increasing infection susceptibility, a particularly concerning issue for patients already immunocompromised by GBM and its treatments. Second, the unique anatomical and physiological features of the central nervous system—specifically the blood–brain barrier, specialized vascular niches, and a tightly regulated immune environment—complicates and limits delivery of targeted therapies to the tumor site and therapeutic specificity, necessitating innovative formulations such as nanoparticles, local delivery, or bispecific approaches that selectively reprogram neutrophils within the CNS. Third, the functional redundancy and plasticity of neutrophils may limit the efficacy of single-agent strategies. Overcoming these barriers may require combinatorial approaches—such as altering NET, integrating neutrophil modulation with immune checkpoint blockade, reversing immunosuppressive polarization, enhancing antigen presentation, radiotherapy, or metabolic reprogramming—alongside strategies to enhance brain penetration, reduce systemic toxicity, and ensure selective targeting within the central nervous system [66].

### Future priorities

Collectively, these gaps highlight several priorities for the field. First, high-resolution, multimodal approaches should be applied to achieve a refined taxonomy of neutrophil subsets in GBM. Second, studies must extend beyond static analyses to capture the temporal evolution of neutrophil states in both patients and models. Third, translational research should focus on developing therapeutic strategies that balance efficacy with safety, ensuring that interventions do not inadvertently compromise essential innate immune functions. Finally, cross-disciplinary collaborations integrating immunology, neuroscience, genomics, and bioengineering will be essential to translate neutrophil biology into clinical benefit for GBM patients.

In summary, while neutrophils are increasingly recognized as central orchestrators of the GBM microenvironment and immune evasion, a deeper and more dynamic understanding is required to harness their potential as therapeutic targets. Addressing these knowledge gaps with innovative technologies and carefully designed translational strategies may ultimately reveal new opportunities to improve outcomes in this devastating disease.

## Conclusion

Neutrophils have emerged as pivotal and multifaceted players in the pathophysiology of GBM, extending far beyond their classical role in innate immunity by exerting diverse and context-dependent effects that shape the TME. Once considered passive bystanders, these cells are now recognized as active orchestrators of tumor growth, angiogenesis, immune suppression, and stem-like cell maintenance, while under certain conditions retaining the capacity for anti-tumor activity. Their remarkable plasticity and ability to engage in extensive crosstalk with other immune and stromal elements highlight their central role in the dynamic ecosystem of GBM. Evidence from clinical and experimental studies underscores the impact of neutrophil infiltration as a biomarker, such as the impact of NLR on predicting patient prognosis.

This evolving understanding has important therapeutic implications. Given their dual capacity to either promote or restrain tumor progression, neutrophils represent a promising yet challenging frontier in GBM immunotherapy. Current immunotherapy approaches for glioblastoma have mainly focused on T cell–centered interventions, but the ability of neutrophils to modulate adaptive immune responses underscores the need to broaden therapeutic perspectives. Combination regimes and emerging therapeutic strategies aimed at modulating neutrophil recruitment, polarization, or effector functions—whether by targeting chemokine signaling, neutrophil extracellular traps, or synergizing with checkpoint blockade—may unlock new opportunities and help overcome current barriers to effective GBM immunotherapy. However, the inherent complexity and context-dependence of neutrophil biology demand careful consideration to avoid unintended consequences of immune modulation.

An integrated research agenda is required to fully harness neutrophils as therapeutic targets or allies. Bridging basic mechanistic insights with translational and clinical studies will be essential to define neutrophil heterogeneity, clarify their temporal and spatial dynamics in GBM, untangle their dualistic roles, and identify actionable targets. Equally critical is translating these insights into rational clinical interventions, guided by robust biomarkers and informed by an appreciation of potential toxicities. By incorporating neutrophils into the broader framework of GBM immunology, future research can advance a more comprehensive understanding of tumor–immune interactions and foster novel strategies to overcome immune evasion. Ultimately, advancing our understanding of neutrophil biology in glioblastoma has the potential to redefine therapeutic paradigms, opening avenues toward more effective and durable treatments for this devastating disease.

## Data Availability

No datasets were generated or analysed during the current study.
